# Prediction method for the truck's fault time in open-pit mines based on exponential smoothing neural network

**DOI:** 10.1038/s41598-023-45675-2

**Published:** 2023-10-30

**Authors:** Wei Liu, Jiayang Sun, Jinbiao Huang, Guangwei Liu, Runcai Bai

**Affiliations:** 1 College of Science, Liaoning Technical University, Fuxin, China; 2 College of Mines, Liaoning Technical University, Fuxin, China; 3 Ningxia Coal Basic Construction Co. Ltd., Yinchuan, China; 4 Liaoning Academy of Mineral Resources Development and Utilization Technology and Equipment, Liaoning Technical University, Fuxin, China

**Keywords:** Energy infrastructure, Applied mathematics, Computer science

## Abstract

The transport truck is one of the important equipment for open-pit mines, and predicting the truck's fault time is of great significance in improving the economic benefits of open-pit mines. In this paper, we discuss the reason for the large prediction error of the exponential smoothing method. Then, we propose a novel nonlinear exponential smoothing method (ESNN) for predicting the truck's fault time, and demonstrate the equivalence between our approach and the neural network structure. Finally, based on the augmented Lagrange function, the solving method of ESNN is proposed. We conduct experiments on real-world datasets and our results demonstrate the effectiveness of ESNN in comparison to existing state-of-the-art methods. Our approach makes it easier for maintenance personnel to predict fault situations in advance and provides a basis for enterprises to develop preventive maintenance plans.

## Introduction

Most open-pit mines in China adopt intermittent systems of electric shovel trucks and semi-continuous mining systems^1^. The transport truck is one of the most important equipment for open-pit mines. In the actual production process, the transport truck has a high fault rate and frequent maintenance due to the poor work environment and improper operation. Meanwhile, maintenance costs are very high^2,3^. The reasonable use and scientific maintenance of trucks can improve the reliability of the open-pit mines process. Therefore, exploring the principle of truck malfunctions and developing scientific maintenance plans is of great significance.

The existing fault maintenance mode of open-pit mine trucks is mainly concentrated in the later stage. Due to the lack of maintenance plans and equipment preparations, the company is unable to provide timely maintenance information, which will cause equipment downtime and affect production. To address this challenge, fault diagnosis technology^4–9^ can reduce mining costs to a certain extent by quickly identifying fault types and accelerating equipment maintenance.

Yang Huan et al.^10^ propose a novel diagnosis method for truck gearboxes based on BP neural networks, and provide criteria for identifying safety hazards in gearboxes. Hu et al.^11^ build a classification model based on truck data and propose a method for predicting truck conditions and performance, which significantly improves the efficiency of trucks. Bai Runcai et al.^12^ propose a method for predicting the fault rate of trucks based on Mallat algorithm and ARMA algorithm. Zhang Yongqiang et al.^13^ propose a novel diagnosis method for motor bearing, which uses RBF neural networks and fuzzy integrals for fault diagnosis.

As a post-processing method, fault diagnosis fails to reduce the fault rate of equipment. However, fault prediction^14–19^ is gradually becoming an effective method for reducing the risk of sudden faults by predicting its future state based on degradation characteristics. In recent years, the rapid development of artificial intelligence technology has provided new ideas for fault prediction. Zhang et al.^20^ build a novel index of performance degradation based on the combination of principal component analysis and support vector data. Li Wei et al.^21^ use the Long Short Term Memory (LSTM) network to predict the future status and the K-Nearest Neighbor (KNN) algorithm to predict fault according to the relationship between the running status and faults. Li Zhijun et al.^22^ propose a novel prediction method of fault based on Dynamic Inner Principal Component Analysis (DIPCA), which predicts three different types of faults. For the problems of fault time, Liu Wei et al.^23^ propose a prediction method with the Metropolis–Hastings (MCMC) algorithm. Zhang Qingliang et al.^24^ use the isometric mapping algorithm to perform feature reduction, and use LSTM to predict the remaining life of automobile gears.

The related work mainly revolves around two perspectives: predicting fault types and predicting equipment's remaining life. However, little research has focused on predicting the truck's fault time in open-pit mines. In this paper, we analyze the principle of the exponential smoothing algorithm^25–28^ and the reasons for the large prediction error of the truck's fault time in open-pit mines. On this basis, we propose a nonlinear exponential smoothing method and construct an equivalent neural network model to estimate its parameters. Our approach can significantly improve the prediction accuracy of the exponential smoothing method. Table 1 shows the summarizing of the related research. Our contributions can be summarized as follows:

**Table 1:** Summarizing the related research.

Index	Related researches
1	A novel method for predicting the time between the faults of trucks is proposed
2	The equivalence between the exponential smoothing model and the neural network model is proved
3	A solving method of our approach is proposed

1. We prove the equivalence between the exponential smoothing model and the neural network;
2. We propose the neural network model based on the nonlinear exponential smoothing method.

The organizational structure of this article is as follows: “Exponential smoothing” discusses the exponential smoothing model; “Neural Network Model Based on Exponential Smoothing (ESNN)” discusses the neural network model based on exponential smoothing; “Results and discussion” presents an empirical evaluation of our algorithm, and “Conclusion” concludes the research work in this paper.

## Method

### Exponential smoothing

Exponential Smoothing (ES) was formally proposed by Brown in 1959, who believed that the time status is stable so that the time series can be reasonably extended. Based on the above assumption, a weighted linear combination of observation data is used to calculate future predictions. The weight decreases exponentially with the further increase of past observations. The smallest weight is associated with the oldest observations.

According to the number of smoothing times, exponential smoothing methods include single exponential smoothing, second exponential smoothing, and third exponential smoothing. Single exponential smoothing is suitable for predicting data without clear trends or seasonal patterns. The second exponential smoothing is suitable for predicting the future value of the data with a linear trend. The third exponential smoothing is appropriate for the data with an obvious seasonal pattern. Due to the lack of clear trends and seasonal patterns in the observed data during equipment faults, single exponential smoothing is chosen as the basis for the algorithm. The future prediction is defined as:

1 $$ \overset{\lower0.5em\hbox{$\smash{\scriptscriptstyle\frown}$}}{y}_{t + 1} = \alpha y_{t} + (1 - \alpha )\overset{\lower0.5em\hbox{$\smash{\scriptscriptstyle\frown}$}}{y}_{t} $$

where $$\overset{\lower0.5em\hbox{$\smash{\scriptscriptstyle\frown}$}}{y}_{t + 1}$$ is the predicted value at the time $$t + 1$$, $$y_{t}$$ is the observed value, $$\overset{\lower0.5em\hbox{$\smash{\scriptscriptstyle\frown}$}}{y}_{t}$$ is the predicted value at the time $$t$$, $$\alpha$$ is the smoothing parameter.

The smoothing parameter is often selected based on experience. However, few works are applied to predict the fault time of equipment. Therefore, there is no empirical value for reference. Besides, as time goes by, the distribution of random variables may change. If $\alpha$ is a fixed value, it will also impact the prediction accuracy.

### Neural network model based on exponential smoothing (ESNN)

The classic exponential smoothing method takes the weighted average of the observed data as the future prediction. Therefore, when the observed values fluctuate within a small range of the average value, the exponential smoothing method presents a small error. However, if the variance of the observed values is large, the single exponential smoothing method may cause significant errors at certain time points.

The analysis shows that the main reason for the higher local error is that the weighted average of the observations is directly used as the prediction result. The problem can be solved by establishing a mapping between the weighted average of the observations and the future prediction. We also use the weighted average value as the independent variable to fit the future prediction.

**Figure 1:**
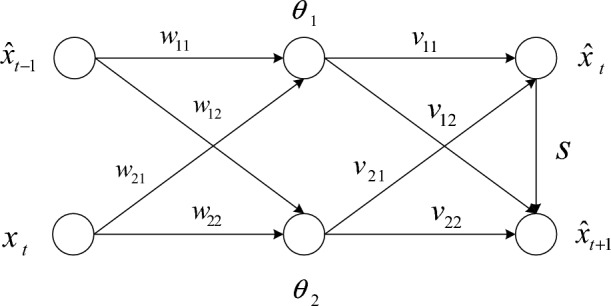
The neural network model is based on exponential smoothing.

Assume that there is a mapping between the weighted average and the future prediction of the observations such that $\hat{x}_{t + 1} = f(\overline{x}_{t + 1} )$, where $\overline{x}_{t + 1} { = }\alpha x_{t} + (1 - \alpha )\hat{x}_{t}$. We build a neural network model equivalent to the above assumptions to establish the mapping and the parameter, as shown in Fig. 1.

From Fig. 1, we can see that:

2 $$ \left\{ {\begin{array}{*{20}l} {\hat{x}_{t} = (w_{11} \hat{x}_{t - 1} + w_{21} x_{t} - \theta_{1} )v_{11} + (w_{12} \hat{x}_{t - 1} + w_{22} x_{t} - \theta_{2} )v_{21} } \hfill \\ {\hat{x}_{t + 1} = (w_{12} \hat{x}_{t - 1} + w_{22} x_{t} - \theta_{2} )v_{22} + (w_{11} \hat{x}_{t - 1} + w_{21} x_{t} - \theta_{1} )v_{12} + \hat{x}_{t} s} \hfill \\ \end{array} } \right. $$

Combining Eq. (2), we can see that:

3 $$ \hat{x}_{t + 1} = (\frac{{w_{12} v_{22} + w_{11} v_{12} }}{{w_{11} v_{11} + w_{12} v_{21} }} + s)\hat{x}_{t} + (w_{22} v_{22} + w_{21} v_{12} - \frac{{w_{21} v_{11} + w_{22} v_{21} }}{{w_{11} v_{11} + w_{12} v_{21} }})x_{t} + \frac{{v_{11} \theta_{1} + v_{21} \theta_{2} }}{{w_{11} v_{11} + w_{12} v_{21} }} $$

If Eq. (3) satisfies the following conditions:

4 $$ \left\{ {\begin{array}{*{20}l} {(\frac{{w_{12} v_{22} + w_{11} v_{12} }}{{w_{11} v_{11} + w_{12} v_{21} }} + s) + (w_{22} v_{22} + w_{21} v_{12} - \frac{{w_{21} v_{11} + w_{22} v_{21} }}{{w_{11} v_{11} + w_{12} v_{21} }}){ = 1}} \hfill \\ {v_{11} \theta_{1} + v_{21} \theta_{2} { = 0}} \hfill \\ \end{array} } \right. $$

Then, this neural network model is equivalent to the single exponential smoothing model. However, if the activation function $g$ is applied to the node $\hat{x}_{t + 1}$.

5 $$ \hat{x}_{t + 1} = g((\frac{{w_{12} v_{22} + w_{11} v_{12} }}{{w_{11} v_{11} + w_{12} v_{21} }} + s)\hat{x}_{t} + (w_{22} v_{22} + w_{21} v_{12} - \frac{{w_{21} v_{11} + w_{22} v_{21} }}{{w_{11} v_{11} + w_{12} v_{21} }})x_{t} ){ + }\frac{{v_{11} \theta_{1} + v_{21} \theta_{2} }}{{w_{11} v_{11} + w_{12} v_{21} }} $$

Equation (5) is the previously defined mapping $\hat{x}_{t + 1} = f(\overline{x}_{t + 1} )$. Training ESNN can solve the parameters in Eq. (5). Assuming the activation function is a hyperbolic tangent function in the node $\hat{x}_{t + 1}$. The hyperbolic sine function has monotonicity and periodicity, which is an activation function. At the same time, it has high complexity and the ability to introduce nonlinearity into neural networks, making it suitable for this paper. The mean square error of ESNN on the training set ${\text{D}}$ ($D = \{ x_{1} ,x_{2} ,...,x_{m} \}$) is:

6 $$ E = \frac{1}{m}\sum\limits_{t = 1}^{m} {(x_{t + 1} - \hat{x}_{t + 1} )^{2} } $$

where,

7 $$ \hat{x}_{t + 1} = \tanh ((\frac{{w_{12} v_{22} + w_{11} v_{12} }}{{w_{11} v_{11} + w_{12} v_{21} }} + s)\hat{x}_{t} + (w_{22} v_{22} + w_{21} v_{12} - \frac{{w_{21} v_{11} + w_{22} v_{21} }}{{w_{11} v_{11} + w_{12} v_{21} }})x_{t} ){ + }\frac{{v_{11} \theta_{1} + v_{21} \theta_{2} }}{{w_{11} v_{11} + w_{12} v_{21} }} $$

Besides, the weights $w_{11} ,w_{12} ,w_{21} ,w_{22} ,v_{11} ,v_{12} ,v_{21} ,v_{22} ,s$ need to satisfy the first equation in Eq. (4). Transform the training of the ESNN model into an optimization process for solving constraint equations.

8 $$ \begin{gathered} \min E \hfill \\ s.t. \, w_{12} v_{22} + w_{11} v_{12} + (w_{11} v_{11} + w_{12} v_{21} )(s - (w_{22} v_{22} + w_{21} v_{12} ) - 1) - w_{21} v_{11} - w_{22} v_{21} = 0 \hfill \\ \end{gathered} $$

Due to the equality constraint of Eq. (8), the backpropagation algorithm will no longer be suitable for solving ESNN models. The constraint in Eq. (8) is nonlinear, so the multiplier penalty function method is introduced to train the ESNN model. First, an augmented Lagrange function from Eq. (6) is constructed by:

9 $$ L({\mathbf{w}},{\mathbf{v}},s,{{\varvec{\uptheta}}},\lambda ,\delta ) = \frac{1}{m}\sum\limits_{t = 1}^{m} {(x_{t + 1} - G({\mathbf{w}},{\mathbf{v}},s,{{\varvec{\uptheta}}}))^{2} } - \lambda F({\mathbf{w}},{\mathbf{v}},s) - \frac{1}{2}\delta F^{2} ({\mathbf{w}},{\mathbf{v}},s) $$

where $${\mathbf{w}}{ = (}w_{11} {,}w_{12} ,w_{21} {,}w_{22} {)}^{T}$$, $${\mathbf{v}}{ = (}v_{11} {,}v_{12} ,v_{21} {,}v_{22} {)}^{T}$$, $${{\varvec{\uptheta}}}_{{1}} { = }(\theta_{1} ,\theta_{2} )^{T}$$.

10 $$ F({\mathbf{w}},{\mathbf{v}},s){ = }w_{12} v_{22} + w_{11} v_{12} + (w_{11} v_{11} + w_{12} v_{21} )(s - (w_{22} v_{22} + w_{21} v_{12} ) - 1) - w_{21} v_{11} - w_{22} v_{21} $$

11 $$ G({\mathbf{w}},{\mathbf{v}},s,{{\varvec{\uptheta}}}){ = }\tanh (P({\mathbf{w}},{\mathbf{v}},s)) + Q({\mathbf{w}},{\mathbf{v}},{{\varvec{\uptheta}}}) $$

where,

12 $$ P({\mathbf{w}},{\mathbf{v}},s){ = }(\frac{{w_{12} v_{22} + w_{11} v_{12} }}{{w_{11} v_{11} + w_{12} v_{21} }} + s)\hat{x}_{t} + (w_{22} v_{22} + w_{21} v_{12} - \frac{{w_{21} v_{11} + w_{22} v_{21} }}{{w_{11} v_{11} + w_{12} v_{21} }})x_{t} $$

13 $$ Q({\mathbf{w}},{\mathbf{v}},{{\varvec{\uptheta}}}){ = }\frac{{v_{11} \theta_{1} + v_{21} \theta_{2} }}{{w_{11} v_{11} + w_{12} v_{21} }} $$

Taking the partial derivatives of Eq. (9) concerning the parameters ${\mathbf{w}},{\mathbf{v}},s,{{\varvec{\uptheta}}}$,

14 $$ \begin{gathered} \frac{{\partial L({\mathbf{w}},{\mathbf{v}},s,{{\varvec{\uptheta}}},\lambda ,\delta )}}{{\partial {\mathbf{w}}}} = \frac{\partial L}{{\partial G}}(\frac{\partial G}{{\partial P}}\frac{\partial P}{{\partial {\mathbf{w}}}} + \frac{\partial G}{{\partial Q}}\frac{\partial Q}{{\partial {\mathbf{w}}}}) + \frac{\partial L}{{\partial F}}\frac{\partial F}{{\partial {\mathbf{w}}}} \hfill \\ \frac{{\partial L({\mathbf{w}},{\mathbf{v}},s,{{\varvec{\uptheta}}},\lambda ,\delta )}}{{\partial {\mathbf{v}}}} = \frac{\partial L}{{\partial G}}(\frac{\partial G}{{\partial P}}\frac{\partial P}{{\partial {\mathbf{v}}}} + \frac{\partial G}{{\partial Q}}\frac{\partial Q}{{\partial {\mathbf{v}}}}) + \frac{\partial L}{{\partial F}}\frac{\partial F}{{\partial {\mathbf{v}}}} \hfill \\ \frac{{\partial L({\mathbf{w}},{\mathbf{v}},s,{{\varvec{\uptheta}}},\lambda ,\delta )}}{\partial s} = \frac{\partial L}{{\partial G}}(\frac{\partial G}{{\partial P}}\frac{\partial P}{{\partial s}} + \frac{\partial G}{{\partial Q}}\frac{\partial Q}{{\partial s}}) + \frac{\partial L}{{\partial F}}\frac{\partial F}{{\partial s}} \hfill \\ \frac{{\partial L({\mathbf{w}},{\mathbf{v}},s,{{\varvec{\uptheta}}},\lambda ,\delta )}}{{\partial {{\varvec{\uptheta}}}}} = \frac{\partial L}{{\partial G}}(\frac{\partial G}{{\partial P}}\frac{\partial P}{{\partial {{\varvec{\uptheta}}}}} + \frac{\partial G}{{\partial Q}}\frac{\partial Q}{{\partial {{\varvec{\uptheta}}}}}) \hfill \\ \end{gathered} $$

where,

15 $$ \begin{array}{l} {\frac{\partial L}{{\partial G}}{ = }\frac{2}{m}\sum\limits_{t = 1}^{m} {(x_{t + 1} - G({\mathbf{w}},{\mathbf{v}},s,{{\varvec{\uptheta}}}))} } \\ {\frac{\partial G}{{\partial P}}{ = }\frac{{1}}{{\cosh (P({\mathbf{w}},{\mathbf{v}},s))}}} \\ {\frac{\partial G}{{\partial Q}} = 1} \\ {\begin{array}{l} {\frac{\partial L}{{\partial F}} = - (\lambda + \delta F({\mathbf{w}},{\mathbf{v}},s))}   \\ \end{array} } \\ \end{array} ,\quad \frac{\partial P}{{\partial {\mathbf{w}}}}{ = }\left( {\begin{array}{l} \begin{gathered} \frac{{\left[ {v_{12} (w_{11} v_{11} + w_{12} v_{21} ) - v_{11} (w_{11} v_{12} + w_{12} v_{22} )} \right]}}{{(w_{11} v_{11} + w_{12} v_{21} )^{2} }}\hat{x}_{t} - \hfill \\ \frac{{\left[ {w_{21} (w_{11} v_{11} + w_{12} v_{21} ) - v_{11} (w_{21} v_{11} + w_{22} v_{21} )} \right]}}{{(w_{11} v_{11} + w_{12} v_{21} )^{2} }}x_{t} \hfill \\ \end{gathered} \\ \begin{gathered} \frac{{\left[ {v_{{{2}2}} (w_{11} v_{11} + w_{12} v_{21} ) - v_{{{2}1}} (w_{{1{2}}} v_{{{2}2}} + w_{{1{1}}} v_{{{1}2}} )} \right]}}{{(w_{11} v_{11} + w_{12} v_{21} )^{2} }}\hat{x}_{t} { + } \hfill \\ \, \frac{{v_{{{2}1}} (w_{21} v_{11} + w_{22} v_{21} )}}{{(w_{11} v_{11} + w_{12} v_{21} )^{2} }}x_{t} \hfill \\ \end{gathered} \\ {(v_{12} - \frac{{v_{11} }}{{w_{11} v_{11} + w_{12} v_{21} }})x_{t} } \\ {(v_{22} - \frac{{v_{21} }}{{w_{11} v_{11} + w_{12} v_{21} }})x_{t} } \\ \end{array} } \right) $$

16 $$ \begin{gathered} \frac{\partial F}{{\partial {\mathbf{w}}}}{ = }\left( {\begin{array}{*{20}c} {v_{12} + s(v_{11} + v_{11} (w_{22} v_{22} + w_{21} v_{12} )) - v_{11} } \\ {v_{22} + s(v_{21} + v_{21} (w_{22} v_{22} + w_{21} v_{12} )) - v_{21} } \\ {v_{12} (w_{11} v_{11} + w_{12} v_{21} )) - v_{11} } \\ {v_{22} (w_{11} v_{11} + w_{12} v_{21} )) - v_{21} } \\ \end{array} } \right) \hfill \\ \frac{\partial Q}{{\partial {\mathbf{w}}}}{ = }\left( {\begin{array}{*{20}c} {\frac{{ - v_{11} (v_{11} \theta_{1} + v_{21} \theta_{2} )}}{{(w_{11} v_{11} + w_{12} v_{21} )^{2} }}} \\ {\frac{{ - v_{21} (v_{11} \theta_{1} + v_{21} \theta_{2} )}}{{(w_{11} v_{11} + w_{12} v_{21} )^{2} }}} \\ 0 \\ 0 \\ \end{array} } \right) \hfill \\ \end{gathered} $$

The partial derivatives of $\frac{{\partial L({\mathbf{w}},{\mathbf{v}},s,{{\varvec{\uptheta}}},\lambda ,\delta )}}{{\partial {\mathbf{v}}}}$, $\frac{{\partial L({\mathbf{w}},{\mathbf{v}},s,{{\varvec{\uptheta}}},\lambda ,\delta )}}{\partial s}$, $\frac{{\partial L({\mathbf{w}},{\mathbf{v}},s,{{\varvec{\uptheta}}},\lambda ,\delta )}}{{\partial {{\varvec{\uptheta}}}}}$ are similar to those of $\frac{{\partial L({\mathbf{w}},{\mathbf{v}},s,{{\varvec{\uptheta}}},\lambda ,\delta )}}{{\partial {\mathbf{w}}}}$. Due to limited space, partial derivatives can not be listed individually.

In Eq. (14), the parameters ${\mathbf{w}},{\mathbf{v}},s$ and ${{\varvec{\uptheta}}}$ are updated in each iteration. The updating formula for parameters $\lambda$ and $\delta$ is defined as:

17 $$ \begin{gathered} \lambda_{k + 1} = \lambda_{k} - \delta_{k} F({\mathbf{w}}_{k} ,{\mathbf{v}}_{k} ,s_{k} ) \hfill \\ \delta_{k + 1} = \rho \delta_{k} \hfill \\ \end{gathered} $$

where $\rho (\rho > 1)$ is the step size. The details of the solution of the ESNN model are described in Algorithm 1.

**Algorithm 1:**
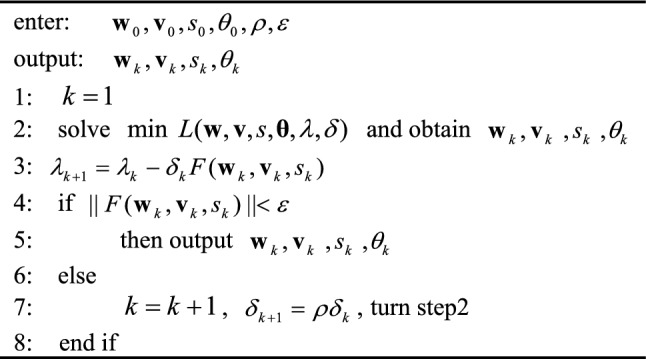
Solve ESNN model.

## Results and discussion

### Test data and environment

The 8-year (2010–2018) maintenance data of the TR100 (Shenhua Baorixile Energy Co., Ltd.) is adopted as the test data. The test data is grouped according to the truck number, and the fault interval time is computed (the next maintenance time minus the previous maintenance time) to obtain the sequence of time between faults. The data has been tested for stationarity, and the results indicate that the test data is a non-stationary sequence.

The ESNN algorithm is implemented in C# language and executed in Visual Studio 2013. All experiments are performed on the same workstation (CPU: E5-2620, memory: 32 GB).

### Settings

For the time series of each truck's fault interval, ESNN and Autoregressive Integrated Moving Average (ARIMA) are used to predict the time between truck faults under four different sliding window sizes (win size). Among them, ARIMA belongs to the classic model in time series analysis, which is widely used due to its high prediction efficiency, small error, and other advantages, and it also conforms to the prediction scenario. Repeat the experiment ten times under each sized sliding window and average the obtained results to produce the final output. In addition, five performance indicators are applied in the experiment: absolute error (AE), relative error(RE), Akaike’s information criterion(AIC), corrected Akaike’s information criterion(AICc), and Schwarz’s Bayesian information criterion(BIC). These five information errors are divided into two categories: traditional metrics (AE and RE) and information criterion metrics (AIC, AICc, BIC). Traditional metrics measure the predictive performance of the model, while the information criteria are used to evaluate the fitting effect of the model.

The testing for stationarity shows that the test data is the non-stationary series. Therefore, the differential order of ARIMA is set as 0. The parameters of ESNN are described in Table 2.

**Table 2:** The parameters of ESNN.

Parameter	ESNN
The number of samples per update	1
Maximum number of iterations	2000
The threshold of convergence	0.1
Learning rate	0.001

In the experiment, we chose sliding window sizes of 6, 7, 8, and 9. They meet the size of the data in this paper and improve prediction accuracy.

### Evaluation

**Figure 2:**
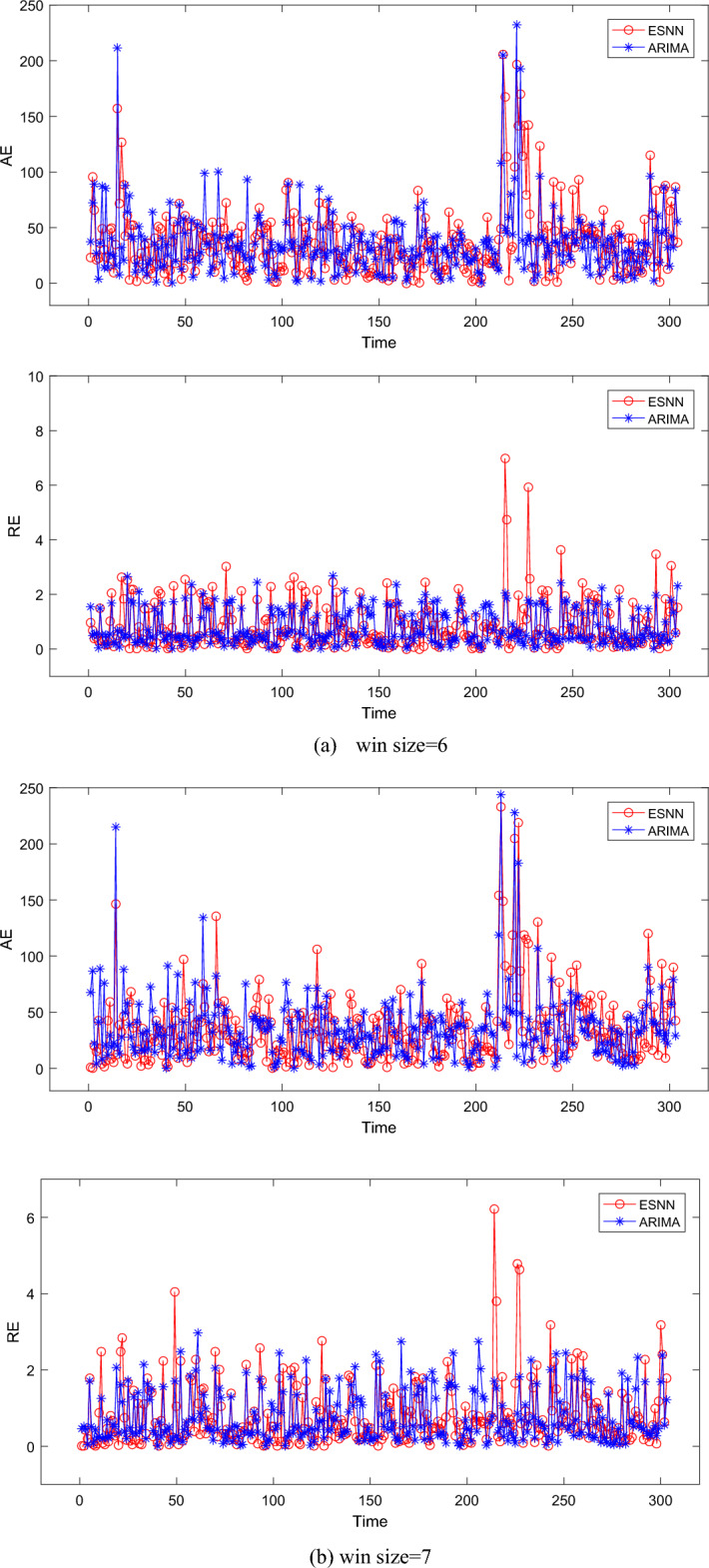

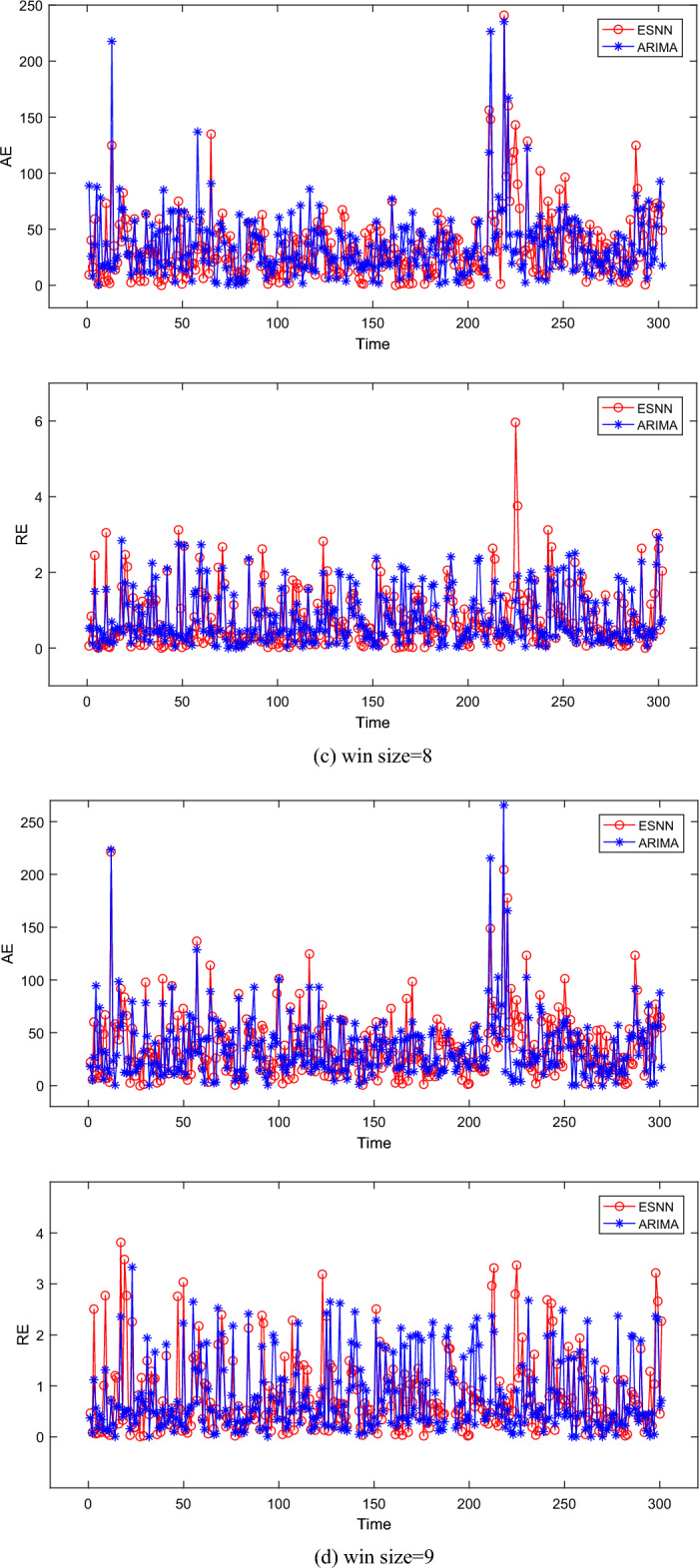
The absolute error and relative error under different moving windows.

Figure 2 shows the AE and RE of four different sliding window sizes. In a summary, it can be observed that the AE of ESNN is smaller than ARIMA, which proves that our approach can improve prediction accuracy. When win size = 6, the AE of ARIMA is slightly higher than ESNN, which is evident in the interval [0, 200]. However, the AE of ARIMA is slightly lower than ESNN; When win size = 7, the AE of ESNN is slightly lower than ARIMA in the interval [0, 50], but ESNN is slightly higher than ARIMA in the interval [250, 280], and the average levels of the AE of ESNN and ARIMA are similar in general; When win size = 8, the AE of ESNN is slightly higher than ARIMA, and it is obvious in the interval [0,60], [80, 120], and [160, 180]. The above phenomenon can be further observed in Table 3. Besides, it can be observed that the AE of ESNN is the lowest when win size = 8. The main reason for the above phenomenon is that the number of data points that ESNN needs to fit increases with the size of the sliding window. Each data point affects the connection weight and neuron threshold of the ESNN in the current sliding window. If the fault interval time of future time points is only related to the fault interval time of the nearest time point, then early time points in the same sliding window will hurt the update of connection weights and neuron thresholds.

**Table 3:** The statistic results of different moving windows.

Sliding window size	ESNN					ARIMA				
	AE	RE	AIC	BIC	AICc	AE	RE	AIC	BIC	AICc
6	37.36	0.81	2384	2431	2385	35.95	0.78	2363	2415	2369
7	36.09	0.78	2374	2420	2376	36.12	0.81	2367	2428	2371
8	34.21	0.76	2334	2372	2335	35.89	0.78	2364	2431	2366
9	37.24	0.81	2368	2405	2367	36.32	0.79	2373	2447	2376
Average	36.23	0.79	2365	2407	2366	36.07	0.79	2367	2430	2371

Table 3 summarizes the results on four types of sliding window sizes for all algorithms. It can be seen that the difference in AE between ESNN and ARIMA is only 0.16, and the RE of ESNN is equal to ARIMA. This indicates that the AE of ESNN is similar to the RE of ARIMA, making it difficult to distinguish which algorithm is better. Therefore, we choose AIC, AICc, and BIC for further analysis. Table 3 shows that the AIC and AICc of ESSN are lower than ARIMA when win size = 8 and win size = 9. The BIC of ESSN is lower than ARIMA when win size = 6. In addition, the average values of AIC, AICc, and BIC in ESNN are the lowest. The gain of ESNN (according to the reduction in AIC, AICc, and BIC) concerning ARIMA is 2, 23, and 5. Overall, ESNN outperforms ARIMA in most indicators. The results show that ESNN has relatively stable prediction error and higher accuracy than ARIMA. This further proves the superiority of ESNN in predicting truck failure time in open-pit mines.

## Conclusion

1. We analyze the principle of the exponential smoothing model and the problems in predicting the truck's fault time, and design a neural network model based on single exponential smoothing method. Compared with existing state-of-the-art methods, ESNN achieves higher accuracy and provides an effective approach for predicting the truck's fault time in open-pit mines.
2. By adjusting the ESNN parameters, we obtain an optimal set of parameters suitable for predicting the fault time of open-pit mines trucks, which provides reliable information for enterprises to develop preventive maintenance plans.

Although the ESNN algorithm has better performance compared to other algorithms, it still has some limitations. For example, the ESNN results are not very prominent for some experimental sliding windows. Due to the structural characteristics of nonlinear exponential smoothing algorithms, the optimized neural network may have a curse of dimensionality in complexity, which makes ESNN unsuitable for predicting long-term fault problems. Therefore, we will conduct further research on these issues in the future, as follows:

1. In the future, we will improve the nonlinear exponential smoothing algorithm to better adapt to the neural network model.
2. In the future, we will reduce the complexity of neural networks to improve prediction accuracy and applicability to higher latitude data.

## Data Availability

The datasets and code used or analyzed during the current study are available from the corresponding author on reasonable request.
